# A method for evaluating the printability of concrete in 3D-printed pavement

**DOI:** 10.1016/j.mex.2025.103450

**Published:** 2025-06-18

**Authors:** Yuping Feng, Guang Yin, Cong Wang, Weizhuo Shi, Yang Zou, Xuhao Wang, Yifeng Ling

**Affiliations:** a School of Civil and Transportation Engineering, Northeast Forestry University, Harbin 150006, China; b School of Qilu Transportation, Shandong University, Jinan 250002, China; c Department of Civil Engineering, University of Nottingham Ningbo China, Taikang Road 199, Ningbo 315100, China; d Department of Civil and Environmental Engineering, University of Auckland, Auckland 1010, New Zealand; e School of Highway, Chang’an University, Xi’an 710064, China

**Keywords:** 3D printed pavement concrete, Printability evaluation, Edge slump, Shape-holding strength, Surface smoothness, A method for evaluating the printability of concrete in 3D-printed pavement

## Abstract

The purpose of this study is to address the limitation that quality control methods of pavement concrete do not account for the printability evaluation of 3D printed pavement concrete. Based on the edge slump test, shape-holding strength test and surface smoothness test, the graded evaluation indicators for the printability of 3D printed pavement concrete were established. To validate the proposed printability evaluation method, a laboratory mini-3D printed paving device was designed. The laboratory results indicate that the edge slump evaluation, shape-holding strength evaluation, and surface smoothness evaluation can quantitatively measure the printability of cement concrete with various mix designs and assess whether the mix proportions of concrete meet the requirements of 3D printed pavement concrete. Additionally, the findings also show that the proposed evaluation method can provide guidance for the optimization of the mix proportions used for 3D printed pavement concrete.

• Edge slump, shape-holding strength, and surface smoothness tests are used to develop graded printability evaluation indicators for 3D printed pavement.
• A laboratory mini 3D printed paver is designed to validate the proposed evaluation method.
• The evaluation method quantitatively measures printability across concrete mix designs.

## Graphical abstract

**Figure:**
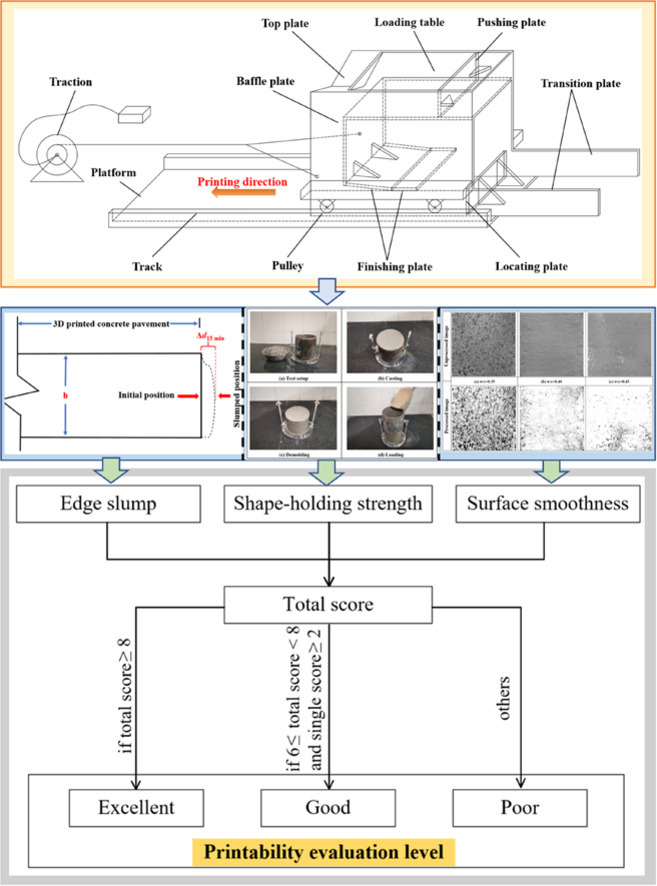

Specifications table

**Table:** 

Subject area:	Engineering
More specific subject area:	*3D printed pavement concrete*
Name of your method:	*A method for evaluating the printability of concrete in 3D-printed pavement*
Name and reference of original method:	*None*
Resource availability:	*Data will be made available on request*

## Background

The construction of traditional cement concrete pavements mainly rely on three-roller or four-roller machinery, in which multiple construction processes (such as installation and removal of formwork, leveling, surface treatment, etc.) collectively constitute the challenges directly leading to an increase in labor demand, a rise in production and construction costs, and an extension of the construction timeline [[1], [2], [3], [4]]. In addition, during the construction process of traditional concrete pavements, manual or mechanical vibration must be used to promote concrete compaction and consolidation, in order to prevent serious quality issues in the concrete pavement, such as the generation of voids and the formation honeycomb structures [5]. However, both manual and mechanical vibration inevitably lead to a high dependence on vibration expertise, which makes quality control of concrete another challenging task [6]. For example, insufficient vibration can result in a large number of voids in the hardened concrete, while excessive vibration can lead to severe inhomogeneities in the concrete, such as aggregate settlement and moisture migration [7,8].

In recent years, 3D printing technology has gained widespread attention in the application of modern concrete structures and shown significant benefits due to the advantages of no vibration, free formwork, low cost and high construction efficiency [[9], [10], [11], [12], [13], [14], [15], [16]]. For example, a study by Ji et al. [17] found that 3D printed concrete can save 60–80 % of construction time and reduce waste generation by more than 60 %. Based on the above advantages and characteristics of 3D printing, combining 3D printing technology with concrete pavement paving can reduce the labor cost, formwork cost and improve the paving efficiency of concrete pavement construction. However, during the process of pavement concrete printing, the concrete needs to have good shape-holding ability and surface smoothness (i.e., printability) without vibration and formwork support to meet the design and construction requirements of the concrete pavements. Evaluating the printability and quality of 3D printed pavement concrete is crucial for the rapid paving and construction of pavements. Although some researchers have employed edge slump test and visual observation to evaluate the shape-holding ability and surface smoothness of concrete [[18], [19], [20]], there is still a lack of research on the quantitative evaluation of the printability for 3D printed pavement concrete.

To overcome this gap, a laboratory mini-3D printed pavement concrete paving equipment was developed in this study. A quantitative evaluation method for printability of 3D-printed pavement concrete was proposed, based on edge slump, shape-retention ability, and surface smoothness. The proposed quantitative evaluation method was then validated through physical testing.

## Method details

### The mini-3D printing paver

**Fig. 1:**
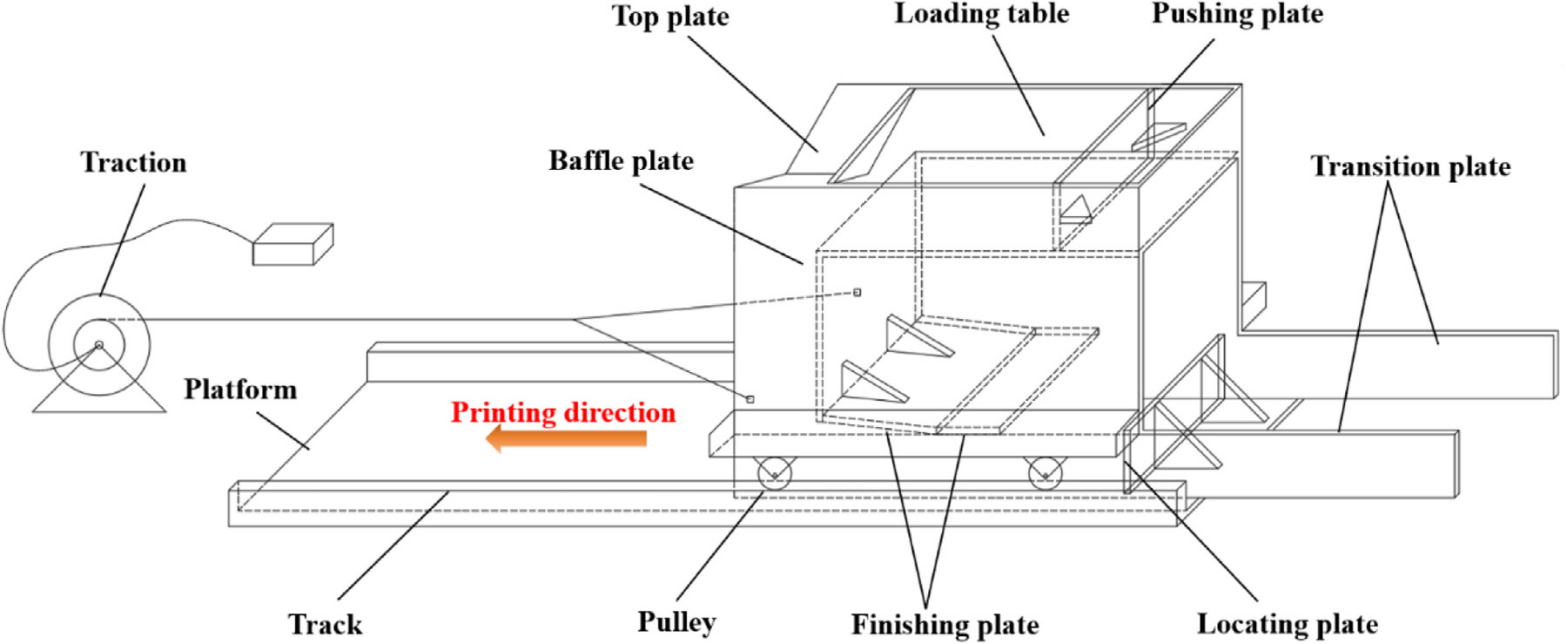
The mini-3D printing paver.

In this work, the developed mini-3D printing paver device consists of a paver, a printing platform, and a traction device, as shown in Fig. 1. The paver having a length of 650 mm, width of 500 mm and height of 450 mm consists of a top plate, baffle plate, finishing plate, loading table, pushing plate, locating plate, transition plate, and pulleys. A box-shaped paver has an opening at the top and bottom, a closed front end, and a printing outlet at the rear. Pulleys are installed on both sides of the bottom, allowing them to move along the track fixed on the printing platform when pulled by the power traction device. The loading table is used to fill the fresh concrete. In paving of 3D printed concrete pavement, freshly mixed concrete fills the space consisting of the top plate, bottom plate, vertical baffle plate, front wall of the box, walls on both sides of the box, and inclined plates. A V-shaped top plate which is lower in the middle and higher on both sides is fixed on the top of the paver box, which is mainly to increase the pressure on both sides of the concrete by increasing the stacking height of the concrete mixture, avoiding the problem of uneven paving of the concrete on the bottom plate due to wall effect on both sides. The finishing plate is composed of the inclined plate and the horizontal plate, and its main function ensures that the concrete surface is squeezed and smoothed during the paving process. The inclined plate has a tilt angle of 6°, with the front end higher than the rear. The combination of the inclined plate and the horizontal plate ensures good smoothness of the concrete surface during the printing process. The inclined plate, horizontal plate, the walls on both sides of the box, and the bottom plate together form the printing outlet of the mini-3D printing paver device.

The width of the paver is 50 mm to 100 mm smaller than that of the track, which is to minimize the impact of the sides of the box bottom plate on the paved concrete. The paver's box is made of plexiglass, which facilitates the observation of the real-time appearance of the concrete pavement during the paving process. The loading table, vertical baffle plate, inclined plate and horizontal plate can be bonded and combined with each other by the strong glue, and the combined structure is fixedly connected by bolts on the side wall of the paver box. The loading platform, vertical baffle plate, inclined plate, and horizontal plate can also be adjusted in angle and height by drilling holes at different heights on the sides of the box and securing them with bolts. Transition plates are installed at the rear sides of the box of the printing paving device. This allows the concrete surface at the starting end to transition from a three-directional stress state to a free state, thereby reducing deformation of the concrete surface caused by stress changes during paving and ensuring stability in the shape of the concrete surface.

The operation of the mini-3D printing paver is as follows: firstly, the bottom plate with tracks is secured to the ground, and the surface of the bottom plate and the tracks are coated with lubricant. Then, the paver device is placed on the bottom plate, where the vertical plate is aligned with the front of the horizontal plate and the pulleys on both sides are fitted to the track. Subsequently, the mixed concrete is poured into the loading table and loaded from the position of the vertical baffle plate until it fills the space formed by the V-shaped top plate, the bottom plate, the vertical baffle plate, the front wall of the box, the walls on both sides of the box, and the inclined plate. Finally, the paver device is driven at a uniform and slow speed by a powered traction system, with concrete mixture continuously supplied from the loading platform. During the paving process, the height of the concrete mixture filling into the vertical baffle plate is maintained at a constant level to ensure that the concrete on the bottom plate is subjected to continuous and constant pressure, thus guaranteeing that the paved concrete is well-formed and compacted. The speed of the paver should be controlled at 2.0–2.5 cm/s. After the concrete mixture has completely slid off the loading table and has been fully extruded through the inclined plate, the paver device should be continuously pulled off the bottom plate without disturbing the extruded concrete during the process. To reduce the impact of water evaporation and cement hydration on the printing quality, the total time consumed from mixing the concrete mixture to completion of the paving should be kept within 20 min.

### Research methodology

3D printed concrete is a rapid construction technique that utilizes the concrete mixture as the ink material to build buildings, with the characteristics of free formwork, high efficiency, and cost savings [10,21]. Applying 3D printing technology to the paving of cement concrete pavement layers can significantly improve construction efficiency and shorten the construction period. 3D printed pavement concrete has extremely high requirements on the concrete mixture. It not only requires the mixture to achieve self-compaction without vibration, but also requires the mixture to achieve self-stability without formwork support. Therefore, evaluating the printability and quality of 3D printed pavement concrete is crucial for promoting 3D printed concrete in rapid repair of pavements and the application in practical engineering projects. In this work, to measure and evaluate the printability of 3D printed pavement concrete, edge slump test, shape-holding strength test, and surface smoothness test were performed on paved concrete pavements.

#### Edge slump test

**Fig. 2:**
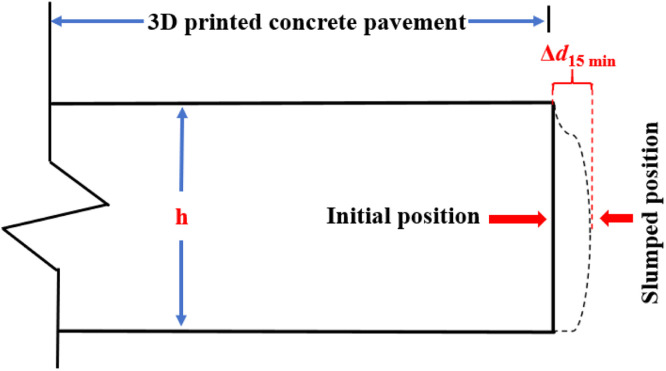
Schematic of edge slump test for 3D printed concrete pavement.

A common performance issue with traditional paver when paving concrete mixture is the edge slump of fresh concrete, which is the edge deformation caused by the placement, consolidation, and extrusion of the fresh concrete from the paver [18]. The conventional slump test is not suitable for evaluating the printability of cement concrete pavement printed using 3D printing technology [22]. To evaluate the edge slump condition of concrete pavements paved by a mini-3D printing paver, a simple laboratory test is conducted to assess the edge slump of pavement concrete. The specific testing procedure is as follows: first, the mixed concrete is placed into the mini-3D printing paver to print a slab-shaped concrete specimen approximately 50 cm long. Then, the thickness of the printed slab-shaped concrete specimen in its initial printed state is measured and the distance between the slump position of the slab-shaped concrete specimen after 15 min and the initial position is recorded. A schematic diagram of the edge slump test for 3D printed concrete pavement is given in Fig. 2. In the edge slump test, the relative edge slump value is used as a measure of the performance of the 3D printed concrete pavement, as shown in Eq. (1). In the edge slump test, relative edge slump is measured at 3 different locations on the same specimen and the mean edge slump value is taken as the final result.

(1) $$\delta =\frac{\Delta d_{15min}}{h}$$

where, δ represents the relative edge slump value (%), $\Delta d_{15\min}$ represents the distance between the slump position of the concrete specimen after 15 min and the initial position (mm), and h represents the thickness of the concrete pavement (mm). Based on the calculation results of the relative edge slump value, the edge slump condition of the 3D printed concrete pavement is categorized into four grades, as shown in Table 1.

**Table 1:** Evaluation grades for edge slump test.

Edge slump grade	Excellent	Good	Average	Poor
δ (%)	0–15	15–25	25–35	35–100

#### Shape-holding strength test

**Fig. 3:**
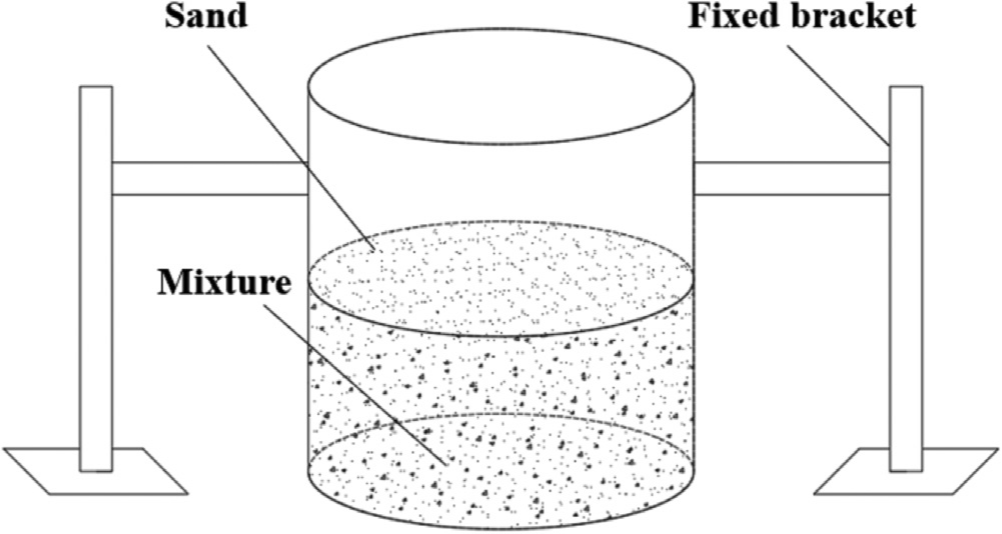
Cylindrical mold for the shape-holding strength test.

**Fig. 4:**
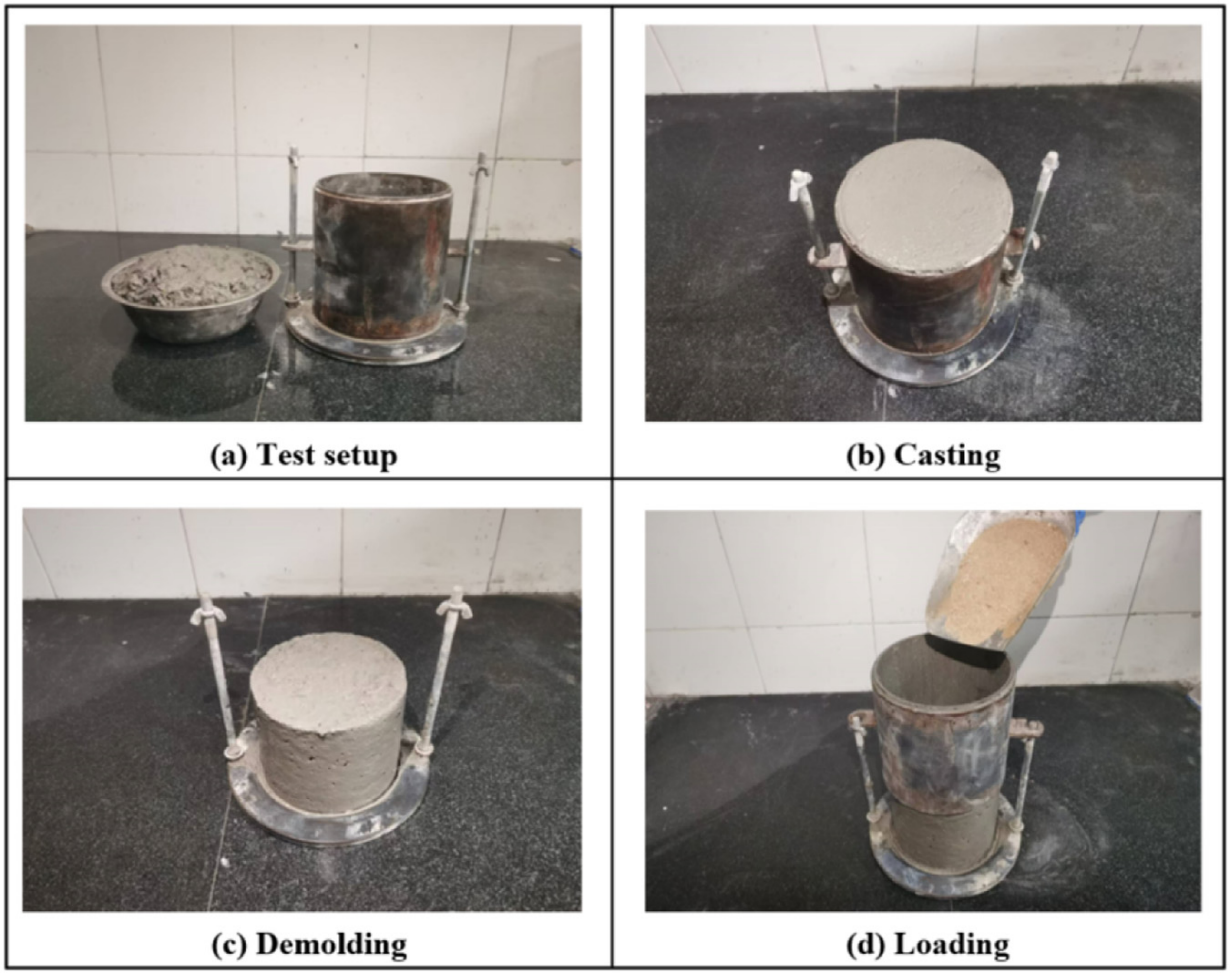
Test procedure for concrete shape-holding strength measurement.

The specific testing procedure for the shape-holding strength test of 3D printed pavement concrete is as follows: a bottomless cylindrical mold with a diameter of 15 cm and a height of 20 cm was employed to cast the concrete mixture, as shown in Fig. 3. During the casting process, the concrete mixture was poured into the mold without any additional consolidation. After completion of casting, the mold was removed vertically, and the shape of the concrete specimen was inspected to determine if it has good self-stability. If the concrete specimen showed no deformation or only minimal deformation within one minute of removing the cylindrical mold, it was considered to exhibit good shape-holding ability. Next, the shape-holding strength test of the concrete specimens was carried out. A cylinder of the same size was fixed on top of the concrete specimen. Subsequently, sand was continuously and uniformly poured into the fixed cylinder at a rate of 50 g/s until the concrete specimen collapsed and the mass of sand poured into the cylinder was recorded. The test procedure for shape-holding strength measurement of concrete specimens is provided in Fig. 4. The calculation of the shape-holding strength is presented in Eq. (2). In the shape-holding strength test, three specimens were fabricated for each set of proportion and the average value of the shape-holding strength of the three specimens was used as the final results. Based on the calculation results of shape-holding strength, the shape-holding ability of the concrete specimens was divided into four levels, as shown in Table 2.

(2) $$\sigma =\frac{mg}{\pi r^{2}}$$

where, σ represents the shape-holding strength (kPa), m represents the Mass of fine sand when the concrete specimen collapses (kg), g represents the gravitational acceleration (9.8 m/s^2^), and r represents the radius of the cylinder (m).

**Table 2:** Evaluation indicators for shape-holding ability.

Shape-holding ability level	Excellent	Good	Average	Poor
Strength (kPa)	> 4	2–4	0.5–2	Immediate collapse or large deformation or < 0.5

#### Surface smoothness test

In the surface smoothness test of 3D printed concrete pavement, in order to more accurately describe and evaluate the surface smoothness of the concrete pavement, MATLAB software was used to process the images of the concrete pavement captured by a camera in an image binarization method. This image processing method is fast and efficient [23]. When capturing images of the concrete pavement, it was necessary to use a camera with good horizontal calibration and environmental adaptability to ensure that the images are both horizontally captured and well lit. When processing images, the captured images were processed using the format of a square with a side length of 50 cm to prevent differences in processing methods from affecting the analysis process. MATLAB applied a threshold value of gray level of 127 to binarize the obtained images, i.e., pixel points with grey level values between 0 and 127 were assigned the value of 0, and pixel points with gray level values between 128 and 255 were assigned the value of 1. 0 and 1 correspond to the black and white in the image, representing the void areas and the compact areas on the surface of the 3D printed concrete pavement, respectively. The black areas were quantified using MATLAB software and the percentage of pore area was calculated according to Eq. (3) to assess the surface smoothness of the 3D printed concrete pavement.

(3) $$p=\frac{A_{1}}{A_{0}}\times 100\%$$

where, p represents the percentage of pore area on the specimen surface (%), A_1_ represents the total area of pores on the surface of the specimen (cm^2^), and A_0_ represents the area of the selected specimen (cm^2^).

Table 3 provides detailed indicators that can be employed in the evaluation of surface smoothness of 3D printed concrete pavement based on the calculated percentage of pore area. It is generally accepted that a satisfactory smoothness should be achieved in order to ensure that the printed concrete is evenly distributed across the surface, with only a minimal number of pores or cracks.

**Table 3:** Detailed evaluation indicators for surface smoothness.

Surface smoothness level	Excellent	Good	Average	Poor
p (%)	0–5	5–15	15–30	30–100

**Fig. 5:**
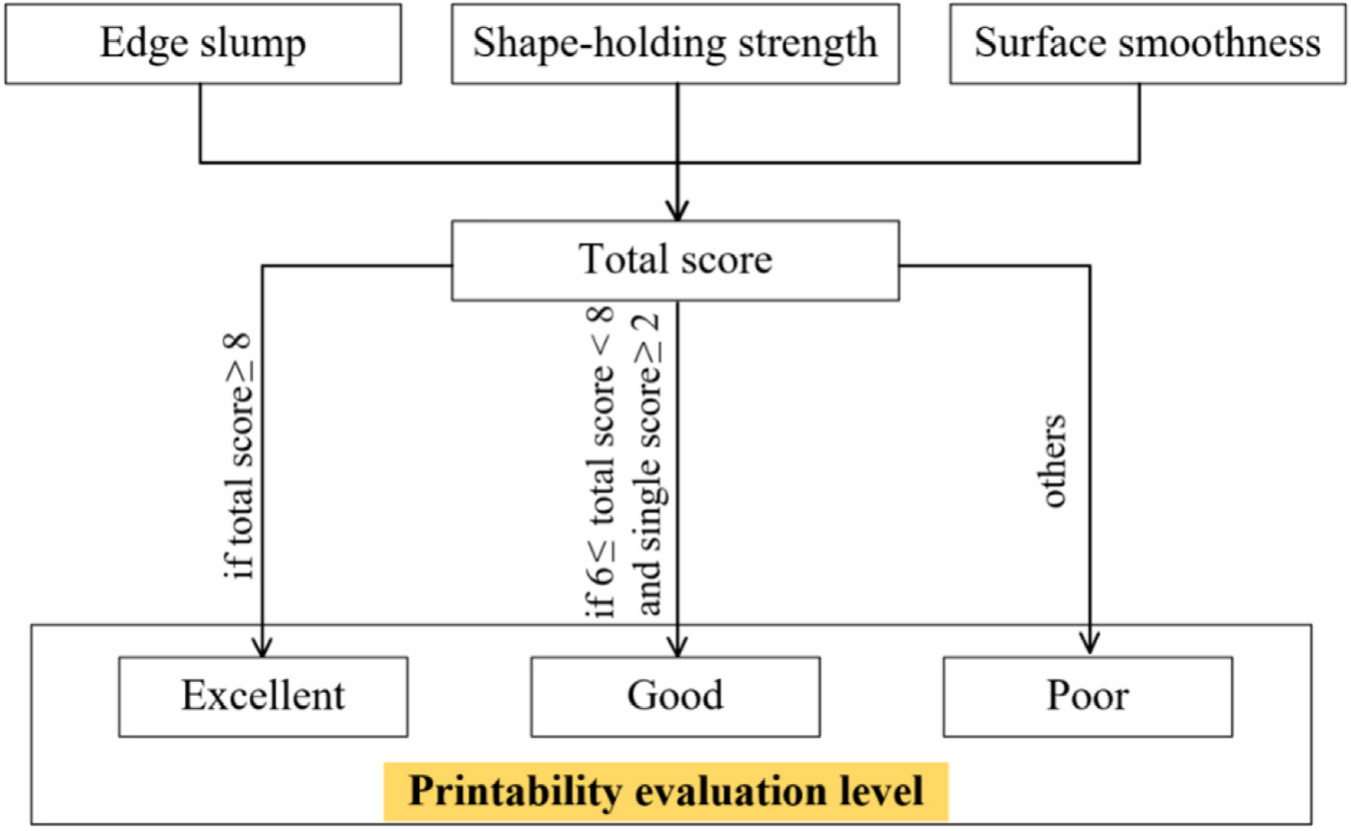
Flowchart for quantitative assessment of printability.

In order to more accurately quantitatively assess the printability of 3D printed pavement concrete, scores were assigned to the graded evaluation levels for different properties, i.e., excellent, good, average, and poor are assigned 3, 2, 1, and 0 points, respectively. When the total score of the concrete mixture is greater than or equal to 8, it is indicated that the concrete mixture at this mix proportion has excellent printability. When the total score of the concrete mixture is greater than or equal to 6 and less than 8 and the score of a single property is greater than or equal to 2, the concrete mixture at this mix proportion has good printability. When the score does not meet the above requirements, it indicates that the concrete mixture does not meet the requirements for printability of 3D printed pavement concrete, and the mix proportion needs to be improved and re-evaluated. The flowchart of quantitative evaluation of 3D printed pavement concrete printability is illustrated in Fig. 5.

## Method validation

### Materials and mix proportions

For 3D printed pavement concrete, different concrete mix proportion designs (e.g., amount of cement, aggregate content, etc.) can affect the printability and the quality of the pavement concrete. Therefore, indoor experiments were conducted using a developed mini-3D printing paver to investigate the impact of mix proportion designs on the printability of 3D-printed pavement concrete in this study. The three mix proportions are prepared as given in Table 4.

**Table 4:** Mix proportions of concrete used for 3D pavement printing (kg/m^3^).

w/c	Cement	Fly ash	Fine aggregate	Coarse aggregate	Water
0.35	340	146	753	961	170
0.40	328	140	849	865	187
0.45	312	134	945	769	201

The water-cement ratios (w/c) of the three mix proportions were 0.35, 0.40, and 0.45, respectively. The main gel material utilized in the work was P.O 42.5 ordinary Portland cement manufactured by Shanshui Cement Group in Shandong Province, China. Its properties were in accordance with the requirements of Chinese standard GB175–2007. In addition, low-calcium fly ash with a density of 2230 kg/m^3^ from Shandong Province, China, was incorporated as a mineral admixture with the objective of reducing the amount of cement, increasing the fluidity of the concrete mix and improving workability. The performance parameters of cement and fly ash are presented in Table 5, Table 6. River sand with a fineness modulus of 2.79 was employed as the fine aggregate, and continuous graded gravel with a particle size ranging from 5 mm to 25 mm was used as the coarse aggregate.

**Table 5:** Chemical composition of cement and fly ash.

Composition (%)	SiO_2_	Al_2_O_3_	Fe_2_O_3_	CaO	MgO	SO_3_	Others
Cement	21.96	4.76	3.68	65.3	2.59	0.30	1.59
Fly ash	50.97	12.19	10.18	6.68	0.95	2.07	5.96

**Table 6:** Physical properties of cement.

Specific surface area (m^2^/kg)	Standard consistency (%)	Dv50 (μm)	Setting time (min)		Compressive strength (MPa)		Flexural strength (MPa)	
			Initial	Finial	3d	28d	3d	28d
358	27.9	17.31	215	270	28.2	50.5	6.4	9.5

### Testing results

#### Edge slump

**Fig. 6:**
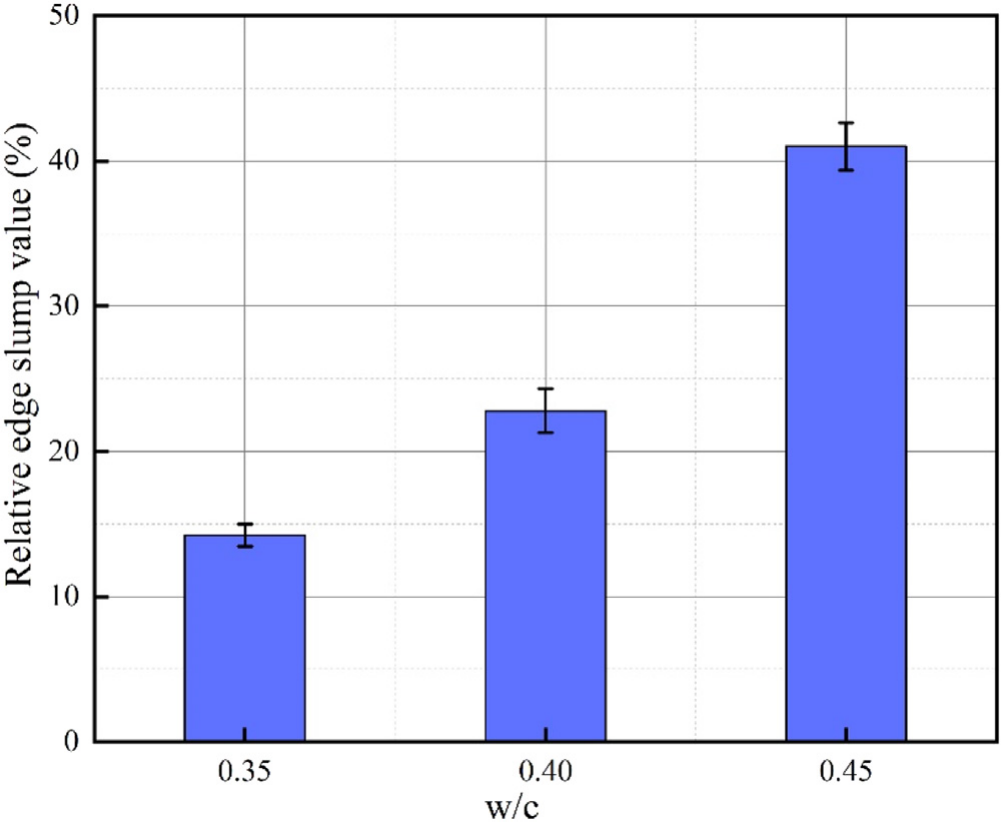
Relative edge slump value of 3D printed pavement concrete.

The relative edge slump values of 3D printed pavement concrete at different mix proportions are illustrated in Fig. 6. The experimental results show that the mix proportion has a significant effect on the edge slump of 3D printed pavement concrete. When the water-cement ratio is 0.35, the relative edge slump of the specimen is less than 15 %, which indicates that the concrete specimen has an excellent edge slump condition. With the increase in the water-cement ratio of the concrete specimens, the relative edge slump of the 3D printed pavement concrete also increases. Specifically, when the water-cement ratio is 0.40, the evaluation grade of the edge slump of the concrete specimen is good. When the water-cement ratio is 0.45, the evaluation grade of the edge slump is poor.

#### Shape-holding strength

**Fig. 7:**
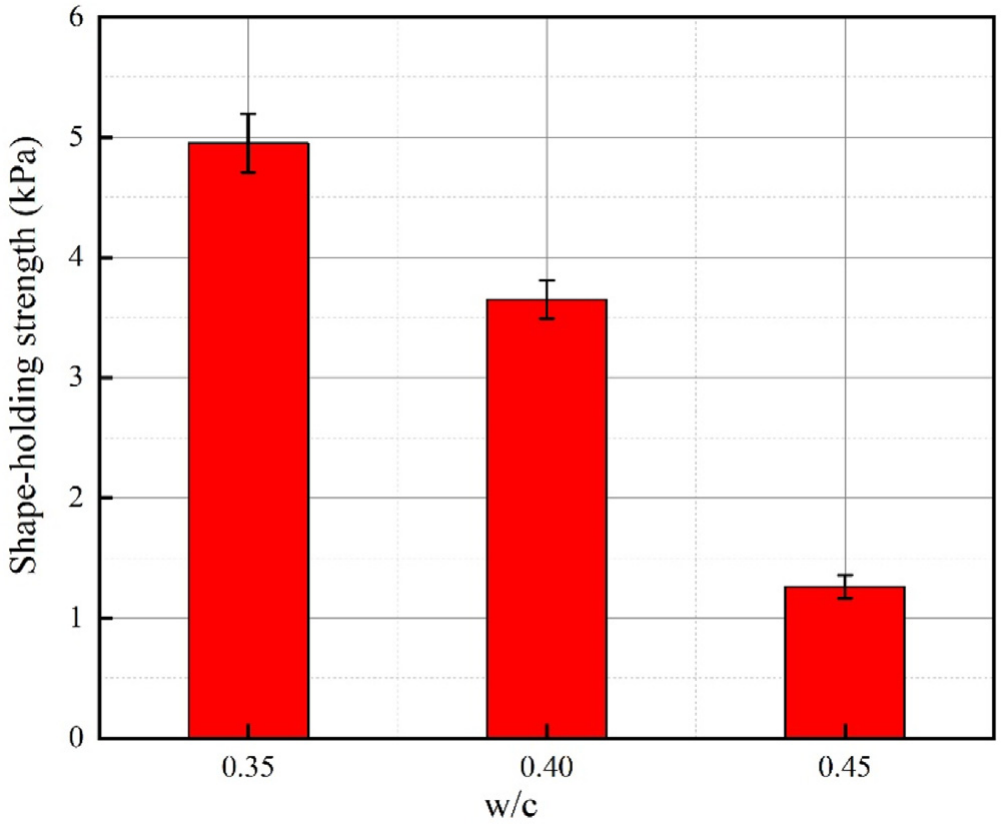
Shape-holding strength of 3D printed pavement concrete.

Fig. 7. presents the shape-holding strength results of the three fresh mixtures measured based on the shape-holding strength test. The shape-holding strength value of the concrete with a water-cement ratio of 0.35 is close to 5 kPa, which is an excellent grade in the shape-holding ability assessment of the three mixtures, indicating that the concrete with a water-cement ratio of 0.35 can achieve sufficient self-stability without the need for formwork support for 3D pavement concrete printing. The shape-holding ability of the mixture with a water-cement ratio of 0.40 is classified as good, and its characteristic is similar to that of the mixture with a water-cement ratio of 0.35, which shows a certain degree of self-stability without significant deformation. As for the concrete mixture with a water-cement ratio of 0.45, it has an average shape-holding strength of 1.26 kPa. When paving concrete with no formwork, serious collapse often occurs, and it is difficult to meet the requirements of concrete pavement due to the lack of good self-stability.

#### Surface smoothness

**Fig. 8:**
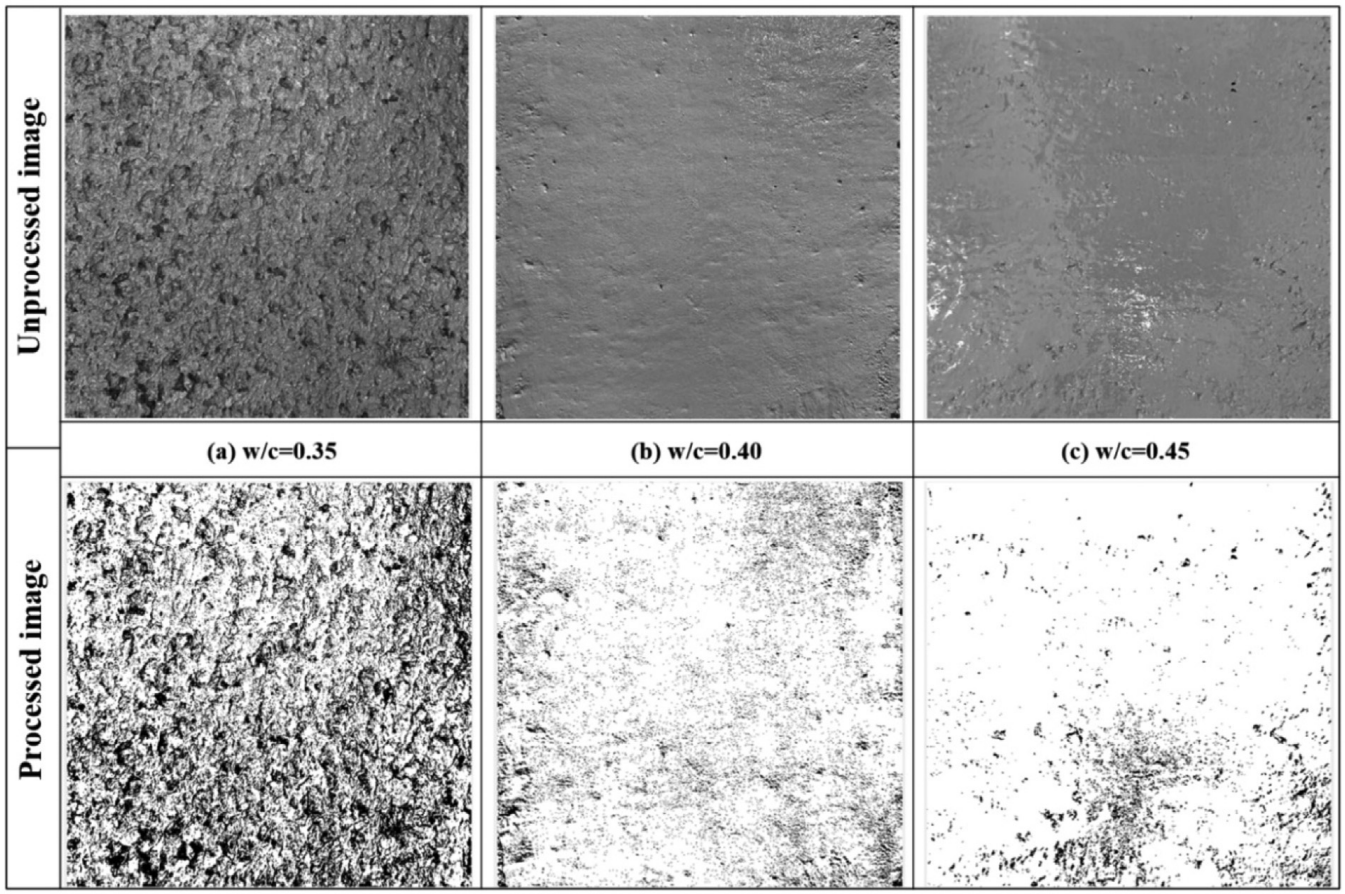
Unprocessed and processed image of 3D printed pavement concrete at different water-cement ratios.

**Fig. 9:**
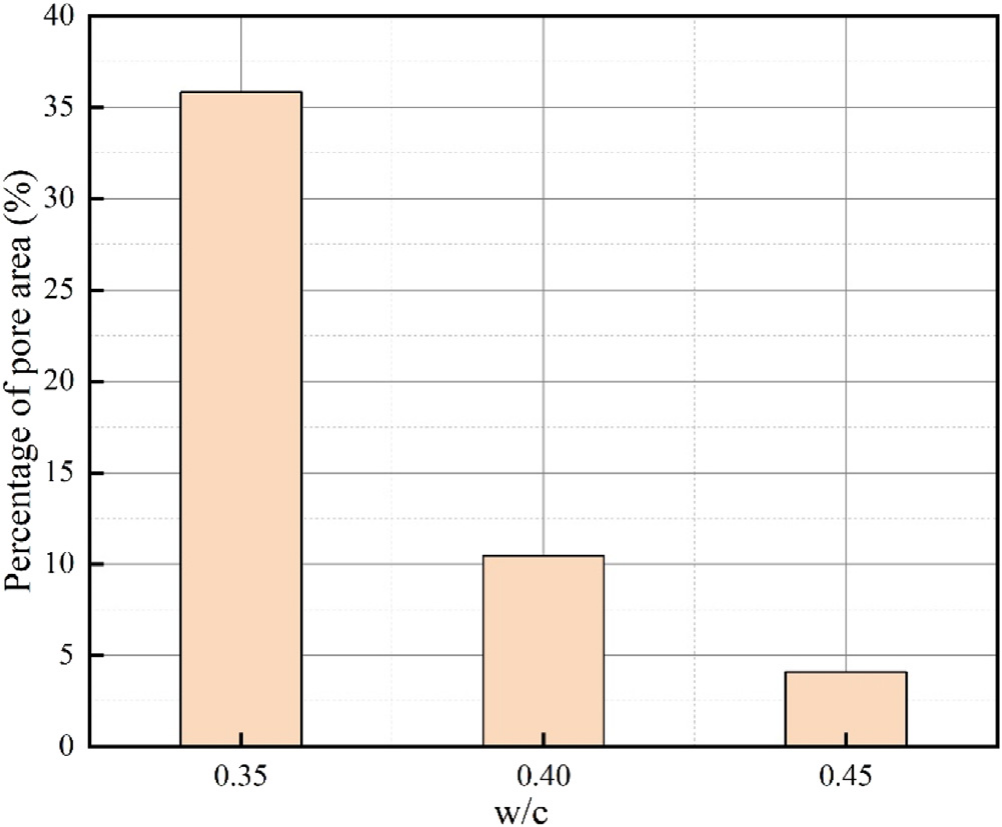
Surface pore area percentage of 3D printed pavement concrete.

The unprocessed and processed surface images of 3D printed pavement concrete at different water-cement ratios are shown in Fig. 8. Fig. 9 demonstrates the results of the surface pore area percentage of 3D printed pavement concrete at various water-cement ratios. The surface pore area percentage of the concrete sample with a water-cement ratio of 0.35 is 35.84 %, which is 3.43 times that of the concrete sample with a water-cement ratio of 0.40. According to the surface smoothness evaluation criteria of 3D printed pavement concrete, the surface smoothness of concrete specimens with a water-cement ratio of 0.35 is recognized as poor. The surface smoothness grade of concrete specimens with a water-cement ratio of 0.40 and 0.45 is good and excellent, respectively. The poor surface smoothness of the 3D printed pavement concrete sample caused by the lower water-cement ratio may be due to the fact that the concrete with a low water-cement ratio contains less water and the flowability of the concrete mixture becomes poor, resulting in the appearance of pores and bubbles on the concrete surface. Additionally, a lower water-cement ratio can also result in that the cement paste cannot fully coat all the aggregates, causing larger particles of aggregate to be exposed on the surface of the concrete, which reduces the smoothness of the concrete surface.

Table 7 provides a comprehensive evaluation of the printability of 3D printed pavement concrete containing different proportions. The evaluation results of the edge slump test, shape-holding strength test and surface smoothness test for the 3D printed pavement concrete show that the concrete specimens with a lower water-cement ratio have excellent edge slump and the strongest shape-holding ability, while the concrete specimens with a higher water-cement ratio have the best surface smoothness. Based on the combined results of the three tests, under the criteria of ensuring good edge slump, strong shape retention, and good surface smoothness for 3D printed pavement concrete, the concrete sample with a water-cement ratio of 0.40 demonstrates good printability.

**Table 7:** Comprehensive evaluation of printability of 3D printed pavement concrete with different proportions.

w/c	Edge slump	Shape-holding strength	Surface smoothness	Total score	Printability evaluation level
0.35	3	3	0	6	Poor
0.40	2	2	2	6	Good
0.45	0	1	3	4	Poor

## Limitations

This evaluation method does not seem to be suitable for concrete with high fluidity, such as self-compacting concrete. For assessing the printability of such materials, future work must incorporate dynamic rheological tests (e.g., l-box, V-funnel) coupled with quantitative analysis of shear-thinning behavior, which will be the focus of our follow-up research.

## Ethics statements

This work did not involve human subjects, animal experiments data, and data collected from social media platforms.

## Supplementary material *and/or* additional information [OPTIONAL]

None

## CRediT authorship contribution statement

**Yuping Feng:** Methodology, Investigation, Formal analysis, Writing – original draft. **Guang Yin:** Investigation, Formal analysis. **Cong Wang:** Conceptualization, Methodology, Investigation, Formal analysis, Writing – review & editing. **Weizhuo Shi:** Formal analysis, Writing – original draft, Funding acquisition, Visualization. **Yang Zou:** Methodology, Writing – review & editing. **Xuhao Wang:** Conceptualization, Resources, Funding acquisition, Writing – review & editing. **Yifeng Ling:** Conceptualization, Methodology, Funding acquisition, Project administration.

## Declaration of competing interest

The authors declare that they have no known competing financial interests or personal relationships that could have appeared to influence the work reported in this paper.

## Data Availability

Data will be made available on request.
